# Gaucher's disease type III C: Unusual cause of intracardiac calcification

**DOI:** 10.4103/0974-2069.43883

**Published:** 2008

**Authors:** Sejal Shah, Amit Misri, Meenakshi Bhat, Sunita Maheshwari

**Affiliations:** Department of Pediatric Cardiology, Narayana Hrudayalaya Institute of Medical Sciences, Bangalore, India; 1 Department of Genetics, Narayana Hrudayalaya Institute of Medical Sciences, Bangalore, India

**Keywords:** Gaucher's disease type III C, intracardiac calcification, oculomotor apraxia

## Abstract

We report a case of intracardiac calcification associated with oculomotor apraxia and corneal deposits in a 12-year-old girl, who presented with dyspnea on exertion, sinusitis, and epistaxis since the age of 6 years. Unusual presentation with multiorgan involvement prompted us to evaluate her in terms of metabolic/storage disorder. The bone marrow aspirate confirmed the diagnosis of Gaucher's disease.

## INTRODUCTION

Gaucher's disease is the most common lipid-storage disorder with an autosomal recessive pattern of inheritance. It is characterized by deposition of glucocerebrosides in various organs due to deficiency of the enzyme glucocerebrosidase. Type III C has been recently identified, and classically, has cardiac valvular manifestations, unlike other forms of Gaucher's disease. We report this rare case wherein the diagnosis was difficult due to unusual clinical manifestations. Presence of intracardiac calcification on echocardiography prompted detailed clinical and laboratory evaluation that eventually led us to the diagnosis of type III C Gaucher's disease.

### CASE REPORT

A 12-year-old girl, born to consanguineous parents, came to the hospital seeking medical treatment for easy fatigability, dyspnea on exertion, and decreased activity for last 6 months. She also had history of recurrent episodes of sinusitis, epistaxis, and involuntary movements of upper limbs. Similar complaints were observed in one of her maternal first cousin sisters, also born to consanguineous parents, aged 8 years. She had normal growth parameters, intellect, and appearance. Clinical examination revealed pes cavus, blood pressure of 100/60 mm/Hg, and oxygen saturation of 100%. Auscultation of the chest revealed coarse crepitations bilaterally. Restricted movements of both eyeballs in specific directions were observed. Cardiac auscultation revealed normal heart sounds with a 2/6 systolic murmur at the apex and left upper sternal border.

**Figure 1:**
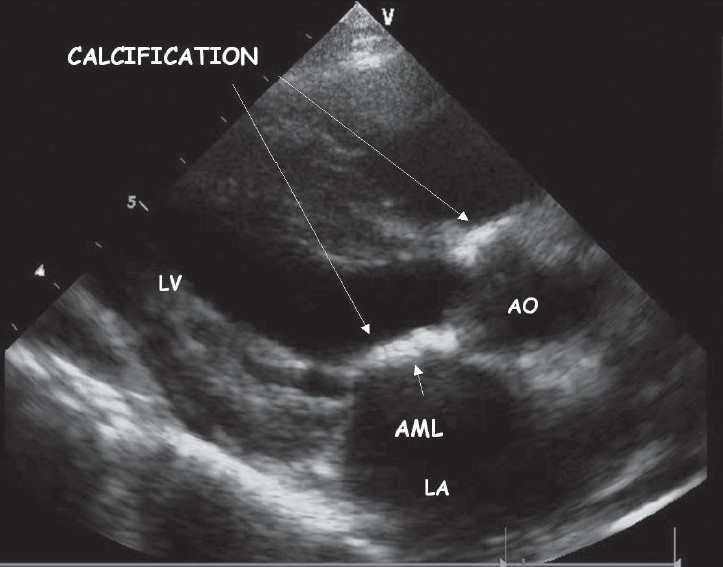
Parasternal long axis view in 2D echocardiogram showing calcification of the aortic root and the anterior leaflet of the mitral valve

**Figure 2:**
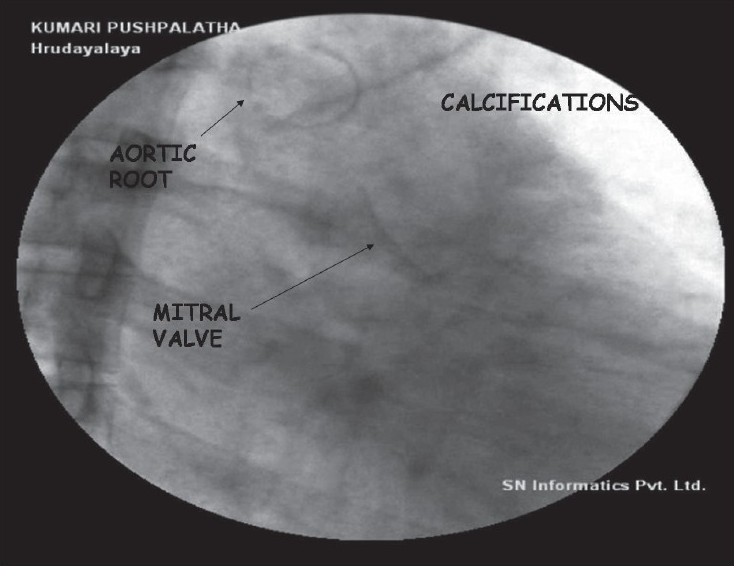
Flouroscopy done in right anterior oblique view confirming the calcifications in the aorto mitral area

**Figure 3:**
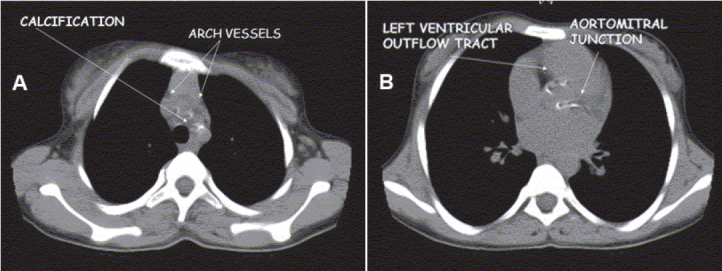
Axial thoracic CT image showing (A) calcifications in the arch vessels, and (B) the aorto mitral area

**Figure 4:**
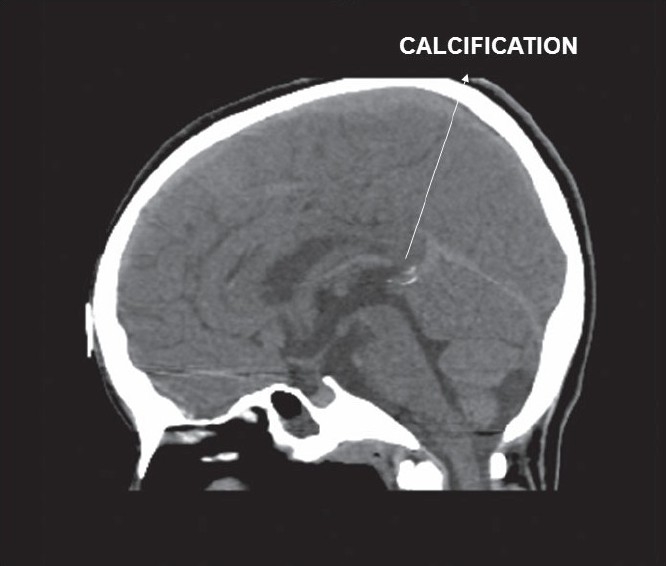
Sagittal CT image of the brain showing calcification in the globus pallidus

**Figure 5:**
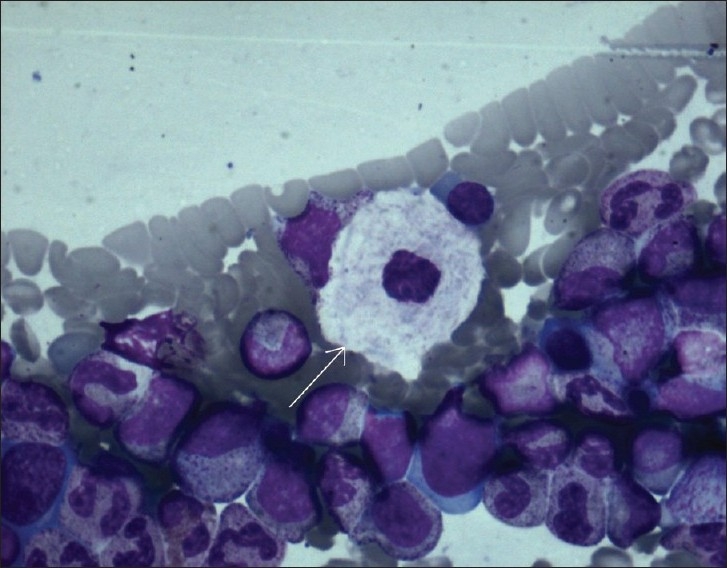
Bone marrow study revealing a large cell with vesicular nucleus and abundant cytoplasm with a wrinkled appearance (Gaucher cell)

Her chest X-ray revealed cardiothoracic ratio of 50% with no evidence of calcification. The lung fields were clear. Echocardiogram showed calcification of endocardium, mitral and aortic valves, and aortic root [Figure 1]. There was moderate mitral regurgitation, mild mitral stenosis, and mild aortic regurgitation. Calcification was also seen surrounding the aortic arch. Fluoroscopy confirmed calcification in the aortomitral area [Figure 2] and around the aortic arch and arch vessels. Thoracic CT scan revealed cardiac calcification [Figure 3A and B], branch pulmonary artery dilatation, and mild compression of left upper bronchus due to dilated left pulmonary artery with left upper lobe and lingular consolidation. To look for other sites of calcification, a head CT scan was done and it showed linear calcification of right globus pallidus [Figure 4] with evidence of sinusitis. Ophthalmic evaluation revealed oculomotor apraxia with corneal dot-like intrastromal deposits. Abdominal sonography showed mild splenomegaly. A bone marrow aspirate performed confirmed the diagnosis of Gaucher's disease [Figure 5]. The cousin sister with similar complaints was also evaluated and found to have intracardiac calcification, oculomotor apraxia, corneal opacities, and splenomegaly. Homozygous D409H mutations were confirmed in both the cousin sisters. The result of enzyme analysis to evaluate fibroblast beta glucocerebrosidase activity is awaited.

## DISCUSSION

Intracardiac calcification is commonly known to occur in rheumatic heart disease, myocardial infarction, pseudohypoparathyroidism, end-stage renal disease on chronic hemodialysis, endomyocardial fibrosis, and pseudoxanthoma elasticum. The heart is not frequently involved in Gaucher's disease.

Type III Gaucher's disease is a late onset, slowly progressive, subacute/chronic disorder presenting with anorexia, respiratory problems, hepatosplenomegaly, seizures, impaired coordination, and disorder of extraocular movements. Most of the patients perish before their thirtieth birthday. Type III A is characterized by myoclonus and dementia, while III B has early onset of isolated horizontal supranuclear gaze palsy with aggressive systemic illness. Type III C is associated with cardiovascular manifestations. Presence of intracardiac calcification in Gaucher's III C has been reported.[1–7] The sites involved are mitral and aortic valves, ascending aorta, aortic arch, and coronary ostia. The calcification in our case had similar distribution, except for the involvement of coronary ostia. Veinot *et al*., first documented the presence of Gaucher cells in the heart valve tissue and proposed a cell-mediated mechanism involving bone matrix proteins and integrins in the pathogenesis of the valvular injury.[5]

The characteristic late onset of presentation with slower progression of complaints in a child born to consanguineous parents, with similar symptoms in a cousin sister, with mild splenomegaly, involuntary movements of the extremities, intracerebral calcifications, oculomotor apraxia with corneal deposits, and cardiovascular calcification made us suspect Gaucher's disease type III C and prompted us to perform a bone marrow aspirate.

In all cases reported till date, there is homozygosity for an asp409his (D409H) mutation in the gene encoding acid beta-glucosidase located on chromosome 1q21.[4,6–9] This mutation was found in both the cousin sisters. Disordered intracellular trafficking of glucocerebrosidase is seen with this mutation. Treatment options include enzyme replacement therapy (ERT) and bone marrow transplantation.[6,9] ERT has been described to improve hematological parameters and organomegaly.[10] Our patient was advised ERT, but could not afford it.

Affected individuals need close monitoring to decide about the need and timing for valve replacement. Surgery is usually offered to those individuals who have clinically and hemodynamically significant valve lesion.[6] Apart from the valve replacement, patients have been subjected to replacement of the ascending aorta and the aortic arch with a Dacron graft when there is a significant calcification resulting in severe narrowing of the aorta. When coronary ostia are involved, one needs to graft the coronary arteries as well.[11]

In conclusion, patients with intracardiac calcification and multiorgan involvement should be investigated for metabolic disorders such as Gaucher's disease as was the case in our patient.
